# Slow Cholinergic Modulation of Spike Probability in Ultra-Fast Time-Coding Sensory Neurons

**DOI:** 10.1523/ENEURO.0186-16.2016

**Published:** 2016-09-26

**Authors:** David Goyer, Stefanie Kurth, Charlène Gillet, Christian Keine, Rudolf Rübsamen, Thomas Kuenzel

**Affiliations:** 1 Institute for Biology II, Department of Zoology/Animal Physiology, RWTH Aachen University, D-52074 Aachen, Germany; 2 Institute of Biology, Faculty of Biosciences, Pharmacy and Psychology, University of Leipzig, D-04103 Leipzig, Germany

**Keywords:** spherical bushy cell, anteroventral cochlear nucleus, gerbil, meriones unguiculatus, olivocochlear bundle, acetylcholine

## Abstract

Sensory processing in the lower auditory pathway is generally considered to be rigid and thus less subject to modulation than central processing. However, in addition to the powerful bottom-up excitation by auditory nerve fibers, the ventral cochlear nucleus also receives efferent cholinergic innervation from both auditory and nonauditory top–down sources. We thus tested the influence of cholinergic modulation on highly precise time-coding neurons in the cochlear nucleus of the Mongolian gerbil. By combining electrophysiological recordings with pharmacological application *in vitro* and *in vivo*, we found 55–72% of spherical bushy cells (SBCs) to be depolarized by carbachol on two time scales, ranging from hundreds of milliseconds to minutes. These effects were mediated by nicotinic and muscarinic acetylcholine receptors, respectively. Pharmacological block of muscarinic receptors hyperpolarized the resting membrane potential, suggesting a novel mechanism of setting the resting membrane potential for SBC. The cholinergic depolarization led to an increase of spike probability in SBCs without compromising the temporal precision of the SBC output *in vitro*. *In vivo*, iontophoretic application of carbachol resulted in an increase in spontaneous SBC activity. The inclusion of cholinergic modulation in an SBC model predicted an expansion of the dynamic range of sound responses and increased temporal acuity. Our results thus suggest of a top–down modulatory system mediated by acetylcholine which influences temporally precise information processing in the lower auditory pathway.

## Significance Statement

Information processing in sensory neural pathways close to the periphery is generally considered to be rigid and therefore less subject to modulation. Here we demonstrate slow cholinergic modulation of information processing in a circuit traditionally seen as a fast and faithful auditory relay station. We combined electrophysiological recordings *in vitro* and *in vivo* with pharmacology and computer modeling to show that the excitability of auditory time-coding neurons is increased by the cholinergic modulation. *In vitro* recordings indicate that the temporal acuity of the time-coding neurons is maintained. This study thus adds a novel component to the understanding of bottom-up-dominated sensory circuitry.

## Introduction

Spherical bushy cells (SBC), the principal neurons of the anterior part of the ventral cochlear nucleus (VCN) of mammals (Brawer et al., 1974), are directly innervated by auditory nerve fibers (ANFs) through giant axosomatic synapses, the endbulbs of Held (Ryugo and Fekete, 1982; Sento and Ryugo, 1989). Afferents from SBCs establish bilaterally converging inputs to both medial nuclei of the superior olivary complex (Cant and Casseday, 1986; Cant and Benson, 2003). Thus, SBCs are often seen as a simple relay. However, a number of studies have shown that SBC activity is not only affected by the excitatory ANF input: the resting membrane potential and action potential probability of SBCs are under the influence of slow inhibitory synaptic inputs (Kuenzel et al., 2011, 2015; Nerlich et al., 2014a; Keine and Rübsamen, 2015) and metabotropic glutamate receptors (Chanda and Xu-Friedman, 2011; Yang and Xu-Friedman, 2015). Additionally, SBC activity is influenced by GABA_B_ receptors (Chanda and Xu-Friedman, 2010) and the dynamics of voltage-activated conductance (Cao et al., 2007; Oertel et al., 2008). Thus, it is obvious that a variety of factors influences the efficacy of information transfer from the ANF to the SBC. Other neuromodulatory influences, such as from the cholinergic system, are also present, but these have been less extensively studied. About three-quarters of cholinergic axons in the VCN are collaterals of the olivocochlear bundle (OCB) fibers originating in the superior olivary complex and thus provide stimulus-driven top–down modulation (Horváth et al., 2000; Guinan, 2006; Kishan et al., 2011). The remaining cholinergic fibers originate in the pontomesencephalic tegmentum (Mellott et al., 2011) and thus carry only indirectly stimulus-driven information. The presence of both nicotinic acetylcholine (ACh) receptors (nAChRs) and muscarinic ACh receptors (mAChRs) has been shown in VCN on a histological level (Happe and Morley, 1998; Yao and Godfrey, 1999a,b; Morley and Happe, 2000; Morley, 2005; Motts et al., 2008; Hamada et al., 2010; Mellott et al., 2011; Schofield et al., 2011). Accordingly, acetylcholine can alter the spike rates of neurons in the VCN (Caspary et al., 1983), and the excitatory effects of ACh were shown for T-stellate cells of the VCN (Fujino and Oertel, 2001; Oertel and Fujino, 2001; Bal et al., 2010). However, recordings from VCN neurons, while simultaneously stimulating the OCB, showed mixed excitatory and inhibitory effects (Mulders et al., 2002, 2003,2009). Thus, the mechanism and functional impact of the cholinergic modulation of VCN neurons, especially concerning the precisely time-coding SBCs, are not yet fully resolved. This would, however, be of great interest since adaptive modulation of temporally precise information processing at the initial stages of the pathway of sound localization could have a large impact on auditory behavior. The present study thus uses both *in vitro* and *in vivo* electrophysiology complemented by histology and *in silico* experiments to scrutinize the mechanisms through which ACh affects the excitability of SBCs in the gerbil VCN.

## Materials and Methods

The *in vitro* experiments were conducted in the laboratories of the Institute for Biology 2 at RWTH Aachen University (Aachen, Germany). All experiments were conducted in accordance with the European Communities Council Directive of 24 November 1986 (86/609/EEC) and were approved by local state authorities (North Rhine-Westphalia State Agency for Nature, Environment and Consumer Protection, Recklinghausen, Germany).

### Slice preparation

Mongolian gerbils (*Meriones unguiculatus*; age range, postnatal day 14 (P14) to P25 of either sex were deeply anesthetized with isoflurane and decapitated, and the brain was quickly dissected in ice-cold cutting buffer containing the following (in mm): 215 sucrose, 10 glucose, 2.5 KCl, 4 Mg_2_Cl 0.1 CaCl_2_, 1.25 NaH_2_PO_4_, 25 NaHCO_3_, 3 C_6_H_12_O_6_ (myoinositol), 2 C_3_H_3_NaO_3_ (sodium pyruvate), and 0.5 C_6_H_8_O_6_ (l-ascorbic acid), bubbled with 95% O_2_ and 5% CO_2_ to a pH of 7.4 (308 mOsm). Coronal or parasagittal slices (150–250 µm) containing the rostral anteroventral cochlear nucleus (AVCN) were cut with a vibrating microtome (VT1200S, Leica Biosystems). The slices were transferred into a holding chamber filled with ACSF containing the following (in mm): 125 NaCl, 2.5 KCl, 1 Mg_2_Cl, 2 CaCl_2_, 1.25 NaH_2_PO_4_, 25 NaHCO_3_, 10 glucose, 3 C_6_H_12_O_6_ (myoinositol), 2 C_3_H_3_NaO_3_ (sodium pyruvate), and 0.5 C_6_H_8_O_6_ (l-ascorbic acid), bubbled with 95% O_2_ and 5% CO_2_ to a pH of 7.4 (314 mOsm). Slices were incubated at room temperature for at least 30–45 min before recordings.

### Electrophysiology

**Figure 1.**
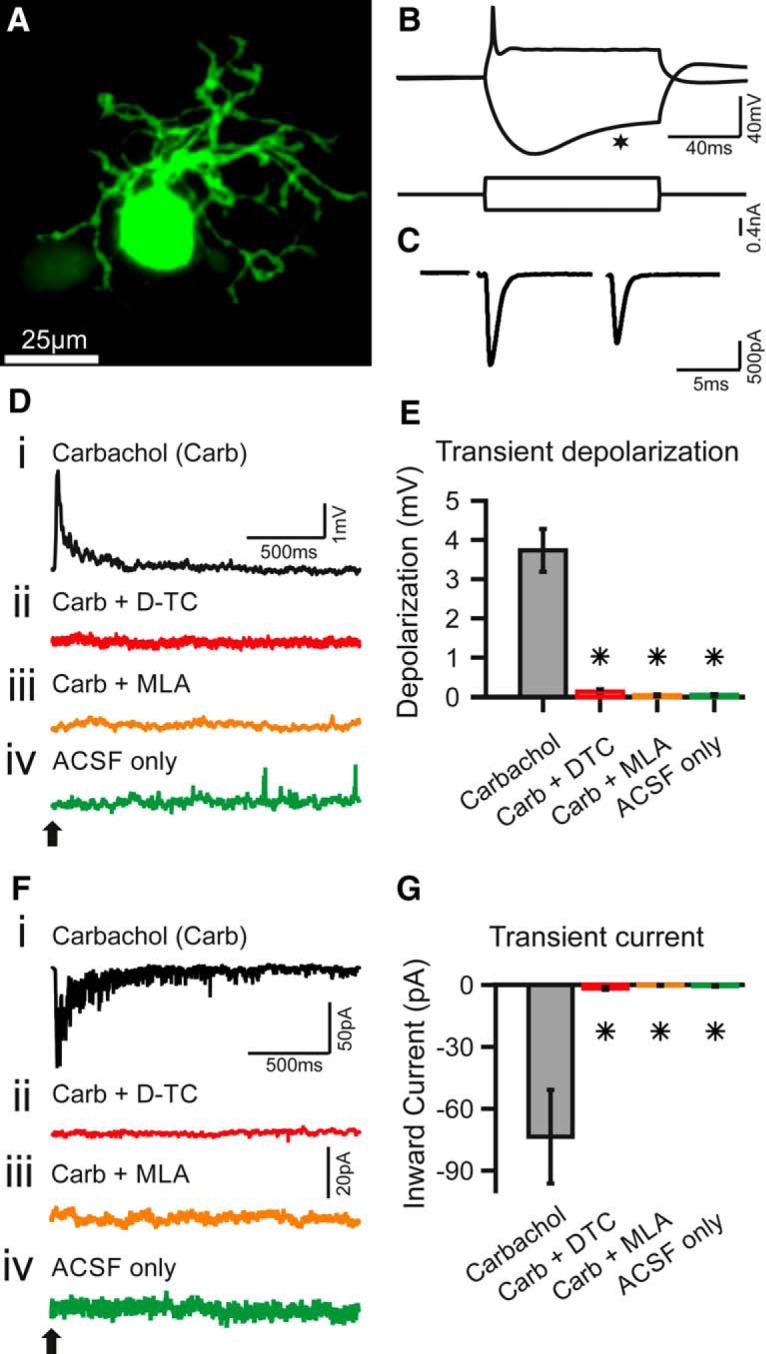
Carbachol application elicits transient depolarization and inward current in SBCs. ***A–C***, Identification and characterization of SBCs. ***A***, Maximum intensity *z*-projection of confocal stacks from a biocytin–streptavidin-labeled SBC. Scale bar, 25 µm. ***B***, Current-clamp recording of a P17 SBC; stimulus is shown below. SBCs typically fire one to two APs upon suprathreshold depolarizing currents and show a pronounced voltage sag upon hyperpolarizing currents (asterisk). Calibration: 40 mV, 40 ms (top); 0.4 nA (bottom). ***C***, The synaptic currents recorded were elicited by electrical stimulation of the auditory nerve in the same SBC as in ***B***; −60 mV holding potential. The succession of two EPSCs shows clear depression, typical for SBCs *in vitro* (stimulus artifacts removed). Calibration: 500 pA and 5 ms. ***D–G***, Transient effects of carbachol-mediated nAChR activation. ***D***, Example traces of current-clamp recordings with puff application of carbachol (Carb; application time is marked by a black arrow). ***Di–iv***, The transient depolarization elicited by the carbachol puff (***i***) was abolished when the slice had been superfused with D-TC, a general nAChR blocker (***ii***); or with MLA, a specific α7 nAChR blocker (***iii***); and with the puffing only the vehicle (ACSF) yielded no effect (***iv***). Calibration: 1 mV and 500 ms (applies to all traces). ***E***, Population data for SBC current-clamp recordings. Asterisks indicate significant difference compared with the carbachol condition (*p* < 0.01, Kruskal–Wallis test with Bonferroni *post hoc* test; carbachol, *n* = 13; Carb+DTC, *n* = 8; Carb+MLA, *n* = 4, ACSF only *n* = 5). ***F***, Example traces of voltage-clamp recordings at −60 mV holding potential with carbachol application (application time marked by a black arrow). ***Fi–iv***, SBCs showed a transient inward current upon carbachol application (***i***), which was abolished under blocker washin of D-TC (***ii***) and MLA (***iii***); and no current was observed upon puff application of the vehicle only (***iv***). Calibration: ***i***, 50 pA and 20 pA; ***ii–iv***, 50 pA and 500 ms. ***G***, Population data for SBC voltage-clamp recordings. Asterisks indicate significant difference compared with carbachol condition (*p* < 0.01, Kruskal–Wallis test with Bonferroni *post hoc* test; carbachol, *n* = 19; Carb+DTC, *n* = 7; Carb+MLA, *n* = 4; ACSF only, *n* = 4).

Slices were placed in a recording chamber (perfused with ACSF at 60–100 ml/h) under a fixed-stage microscope with infrared differential interference contrast (IR-DIC) and fluorescent imaging (Eclipse FN-1 microscope equipped with DS-Qi1MC camera and DC-U3 camera controller, Nikon Instruments). SBCs were patched under visual control with a EPC10 USB double-patch clamp amplifier, controlled with PATCHMASTER software (HEKA Elektronik Dr. Schulze GmbH). Recordings were low-pass filtered at 2.9 kHz and sampled at 50 kHz. Data were analyzed with software custom-written in MATLAB (MathWorks GmbH). All data are presented without correcting the junction potential, which was estimated to be −11 mV. To block the GABAergic/glycinergic inhibitory inputs on SBCs, recordings were performed in the presence of 1 µm strychnine (Sigma-Aldrich Germany), 10 µm gabazine (Abcam UK) and 2 µm CGP-55845 (Biotechne). Recording pipettes were pulled from 0.86/1.5 mm (inner/outer diameter) borosilicate glass filament electrodes (Science Products GmbH) with a horizontal DMZ Universal Puller (Zeitz Instruments) to have a resistance of 45 MΩ when filled with the recording solution. Pipettes were filled directly before recordings with a patch solution containing the following (in mm): 100 K-gluconate, 40 KCl, 0.1 CaCl_2_, 10 HEPES, 1.1 EGTA, 2 Mg-ATP, 0.4 GTP, 0.1 Alexa Fluor 488 hydrazide (Thermo Fischer Scientific), 3 mg/ml biocytin (Thermo Fischer Scientific) and adjusted to a pH of 7.2 with 1 m KOH (280 mOsm). The pharmacological agents and concentrations used to isolate the cholinergic effects were as follows: 50 µm d-tubocurarine (D-TC; Sigma-Aldrich Germany); 20 nm methyllycaconitine (MLA; Sigma-Aldrich Germany), 2 µm atropine (AT; Sigma-Aldrich Germany) and 100 nm tolterodine (Tol; Biotechne). Recordings (Fig. 1; see also Figs. 3, 5) were performed at room temperature (∼23°C). To test for temperature effects, additional recordings (see Fig. 4) were performed at physiological temperature (37°C), using a custom-built, heated slice chamber. In these experiments, the changes of the resting membrane potential (RMP) and membrane resistance (*R*_m_) upon washin of pharmacological agents was monitored for at least 20 min. This was achieved by calculating the linear fits to subthreshold current–voltage relations measured every 30 s (hyperpolarizing and depolarizing current steps of −100 to +100 pA amplitude were injected).

### Pharmacological puff application and synaptic stimulation

For cholinergic stimulation we used carbachol (carbamylcholine chloride, Sigma-Aldrich Germany), a stable acetylcholine analog. Carbachol was diluted in ACSF to a final concentration of 500 µm and filled into glass electrodes with a 3-4 µm tip opening. Carbachol was applied to the patched SBCs with a 2 ms pressure puff through a Picospritzer 2 (General Valve Corporation/Parker Hannifin Precision Fluid Systems) at 6-8 psi, yielding a restricted area of effect and a short duration of delivery of <100 ms. The area and duration of the carbachol puff application were visually adjusted with the aid of fluorescent dye (Alexa Fluor 488, Sigma-Aldrich Germany) in the pipet solution. This method has been successfully used for precisely targeted pressure application (Ko et al., 2016), although some spillover of low concentrations of the compound to neighboring cells cannot be excluded. The Picospritzer was triggered by a preprogrammed recording routine in the HEKA PATCHMASTER software. This routine consisted of 10 recording sweeps, each one with a carbachol puff at the beginning, followed by a 10 s recording span, adding up to 100 s of recording. This allowed measuring of the immediate effects as well as long-lasting effects on the SBC. Weighted time constants of the decay of the cholinergic responses were calculated by fitting double-exponential functions. The weighted time constant was then calculated as $\tau_{w}=(A_{fast}\cdot \;\tau_{fast}+\;A_{slow}\;\cdot \;\tau_{slow})/(A_{fast}+A_{slow}),\;$where *A*_fast_ and *A*_slow_ are amplitudes at *t* = 0, and τ_fast_ and τ_slow_ are the fast and slow time constants, respectively. Recordings were considered for further analysis if the fitting algorithm achieved a goodness of fit (*r* ^2^) >0.8. This criterion resulted in omitting 10 cells that showed depolarization or inward currents of unclear waveform. For synaptic stimulation, a 75 µm bipolar tungsten electrode (MicroProbe Inc.) of 1.5 MΩ impedance was placed in the auditory nerve root (ANR). Auditory nerve fibers were electrically stimulated with monopolar pulses generated by an Iso-Flex Stimulus Isolator (A.M.P.I.), which was triggered by the PATCHMASTER software. A minimal stimulation protocol was used to activate single endbulb of Held inputs. Minimal stimulus intensities were determined for each SBC (range, 5–40 V), and all further experiments were performed at 110% threshold intensity. Sets of *in vivo*-like (IVL) stimulus protocols were generated off-line by using the spike time output of an auditory periphery model (Zilany et al., 2014) as the stimulus arrival times (high-spontaneous rate, cat ANF). One IVL sweep consisted of 200 ms spontaneous activity and 200 ms sound-driven activity [250 Hz, 40 dB sound pressure level (SPL)]. A complete experiment consisted of 25 sweeps representing statistically independent draws of spike trains from the model. Sets of stimuli were identical for controls and treatment conditions. This allowed for comparison of the mean spike probability (P_AP_), mean spike jitter, and the *in vitro* vector strength (VS) of the cell. Pulse trains were applied by the PATCHMASTER software with 3 s intervals between the individual sweeps. To calculate the spike probability of the patched SBCs, only the ratio of successful synaptic events was used (i.e., EPSPs and action potentials). Complete failures (i.e., lack of a detectable event after the stimulus possibly due to subthreshold stimulation) were omitted from the analysis.

### Fluorescence immunohistochemistry

Gerbils in the age range P18 to P31 were killed with an overdose of >150 mg/kg body weight ketamine (Ceva Tiergesundheit GmbH) and then transcardially perfused with ice-cold phosphate buffer (PB) and 4% paraformaldehyde (PFA) in PB. The brain was removed and fixed by immersion in 4% PFA in PB overnight. After fixation, the brains were successively transferred into 10% and 30% sucrose solution for cryoprotection. Brains were embedded in Tissue-Tek (Sakura Finetek), and 30 µm sections were cut either in the coronal or sagittal plane on a cryotome (CM3050S, Leica Biosystems). The sections were collected on gelatinized slides. Double immunohistochemical staining was performed as follows: the sections were rinsed with PBS and incubated with blocking solution [4% normal horse serum (NHS), 0.4% Triton X-100, and 1% bovine serum-albumin (BSA) in PBS)] for 3 h at room temperature. Next, the sections were incubated with a primary antibody solution (1% NHS, 0.3% Triton X-100, and 1% BSA in PBS) for 24 h at 4°C. After rinsing with washing solution (0.02% Triton X-100 and 0.25% BSA in PBS), sections were incubated with secondary antibody solution (0.02% Triton X-100 and 1% BSA in PBS). The sections were rinsed with 0.25% BSA in PBS followed by a nuclear staining with 4′,6′-diamidino-2-phenylindole dihydrochloride (DAPI). Finally, the sections were coverslipped with Fluoprep (bioMérieux), sealed against exsiccation, and stored in the dark at 4°C until analysis with a laser-scanning confocal microscope (TCS SP2, Leica Microsystems). The antibodies and conjugates used were as follows: anti-calretinin (CR; 2 µg/ml; catalog #AB1550, lot #2430339, Merck Millipore); anti-vesicular acetylcholine transporter (VAChT; 3 µg/ml; catalog #139103, Synaptic Systems); and anti-choline acetyltransferase (ChAT; 5 µg/ml; catalog #AB143, lot #2167141, Merck Millipore). All three primary antibodies are listed in the JCN antibody database (http://onlinelibrary.wiley.com/journal/10.1002/(ISSN)1096-9861/homepage/other_resources.htm#AntibodyDatabase) as validated for specificity. The matching secondary antibodies were Alexa Fluor 488 and Alexa Fluor 546 (diluted 1:500; catalog #A11055/A10040, lot #1627966/1640319, Thermo Fisher Scientific). The Alexa Fluor 555-conjugated α-bungarotoxin (BTX; 2 µg/ml; catalog #B35451, Thermo Fisher Scientific) was added to the secondary antibody solution.

### Biocytin–streptavidin labeling of patched SBCs

To identify the patched cells *post hoc* as SBCs, the recorded cells were filled with biocytin (Thermo Fisher Scientific) added to the patch pipette solution. Afterward, the slices were fixed in 4% PFA in PB overnight, then washed six times in PBS for 5 min each, followed by six washing steps with 0.3% Triton X-100 in PBS for 5 min each. The slices were then incubated with streptavidin solution (0.1% Triton X-100, 1% BSA in PBS, 1:800 Alexa Fluor dye streptavidin conjugates; catalog #S11223, Thermo Fisher Scientific) for 2.5 h at room temperature followed by 6× 5 min washes with 0.3% Triton X-100 in Tris-buffered saline (TBS) followed by 3× washes with TBS only. Additionally, nuclear staining with DAPI was performed. The slices were collected on coverslips (24 × 60 mm), which were pasted up with a 15 × 15 mm SecureSeal Adhehsive Sheet (Grace Bio-Labs) 240 µm in thickness and covered with a drop of Fluoprep (bioMérieux). The marked cells were then analyzed with a laser-scanning confocal microscope (TCS SP2, Leica Microsystems).

### *In vivo* recordings

All *in vivo* experiments were performed at the Neurobiology Laboratories at the Institute of Biology of the University of Leipzig, were approved by the Saxonian District Government, Leipzig (TVV 06/09), and were conducted according to the European Communities Council Directive (86/609/EEC). Eighteen Mongolian gerbils between the ages of 22 and 38 d were used. Prior to the experiment, animals were deeply anesthetized with an intraperitoneal injection of a mixture of ketamine hydrochloride (140 µg/g body weight; Ketamin, Ratiopharm) and xylazine (3 µg/g body weight; Rompun, Bayer). During the experiment, subcutaneous injections of about one-third of the initial dose were applied hourly to keep the animal in an areflexic state, indicated by the absence of the limb withdrawal reflex. Pre-experimental surgery was performed as described previously (Keine and Rübsamen, 2015). In brief, the skull of the animal was exposed, and two holes were drilled; the first hole, 1.8-2.0 mm caudal from the lambda point for the reference electrode, and the second, 1.5 mm lateral to the former for the recordings electrode. The animal was fixed in a prone position using a brass headpost glued to the skull of the animal. Glass micropipettes (GB200F-10, Science Products) were filled with 3 m KCl (impedance, 5–8 MΩ) and lowered into the rostral AVCN. For iontophoretic application, three-barrel piggyback electrodes (Havey and Caspary, 1980; Dehmel et al., 2010) were mounted to the recording electrode and filled with glycine (100 mm in ACSF, pH 6, positive control; Sigma-Aldrich) and carbachol hydrochloride (5-500 mm in ACSF, pH 6; Sigma-Aldrich), respectively. A backing current for each barrel was used (−15 nA), and the third barrel was filled with 1 m sodium acetate and served for current balancing. In a subset of experiments, four-barrel electrodes were used, and the fourth barrel filled with ACSF, omitting glycine and carbachol, which served as a negative control. Drugs were applied iontophoretically (EPMS 07; npi electronic) with increasing current steps (+0 to +100 nA; automatic capacitance compensation was applied), while the spontaneous activity of the neuron was monitored. The rostral pole of the AVCN was targeted considering the tonotopic organization of the nucleus (Kopp-Scheinpflug et al., 2002; Dehmel et al., 2010), and SBCs were identified by their characteristic complex waveform (Pfeiffer, 1966; Winter and Palmer, 1990; Englitz et al., 2009; Kuenzel et al., 2011; Keine and Rübsamen, 2015). Extracellular voltage signals were digitized at a sampling rate of 97.7 kHz (24 bit, RP2.1, Tucker-Davis Technologies), band-pass filtered between 50 Hz and 5 kHz, stored, and analyzed using custom-written software in MATLAB (version 8.5, MathWorks).

### Spherical bushy cell model

Simulations were performed as previously published (Kuenzel et al., 2011, 2015; Nerlich et al., 2014a) with NEURON (Hines and Carnevale, 1997, 2000; Hines et al., 2009) using software custom-written in Python version 2.7 under Linux x86_64 for simulation control and analysis. Properties of ionic conductances and membrane biophysical properties of the SBC model were set to match previously published models (Rothman and Manis, 2003), except the sodium channel model we used, which was published earlier (Rothman et al., 1993). Briefly, a somatic compartment, an axon first segment containing all voltage-activated sodium conductance, and a stretch of passive axons were included in the model. Reversal potential for the leak conductance (gLeak total, 14.5 nS) was set to −65 mV. Voltage-activated ion channel conductances were as follows: Na_v_ (voltage-gated sodium), 1000 nS; LVA-K (low-threshold voltage-activated potassium), 200 nS; *I*_h_ (hyperpolarization-activated cation current), 40 nS [reversal potential (E_rev_), −43 mV]; and HVA-K (high-threshold voltage-activated potassium), 175 nS. Basic somatic parameters of the model SBC, therefore, resulted in a total membrane capacitance of 20.1 pF, a total input resistance (at rest) of 69.2 MΩ, and a resting membrane potential of −65.1 mV. All simulations were run at a temporal resolution of 10 µs. A model of the endbulb of Held giant terminal and an inhibitory synaptic input (representing the sum of GABA/glycinergic inputs to the SBC) were connected to the somatic compartment. Synapses were modeled as conductance point sources [excitation, 55 nS (E_rev_, 0 mV); inhibition, 24 nS (E_rev_, −75 mV)], and synaptic dynamics were included as a Gaussian distribution of excitatory postsynaptic conductance (EPSG) amplitudes for the endbulb of Held (55 ± 9 nS; Nerlich et al., 2014a) and as complex rate-dependent plasticity for the inhibitory input (Nerlich et al., 2014a,b). For some simulations, inhibitory conductance was set to 0 nS, and excitatory conductance was reduced accordingly to again match the failure rates of endbulb of Held synapses observed *in vivo*. Conductance waveforms for the synaptic mechanisms were generated by convolving EPSG template waveforms with the spike arrival times of the respective inputs at the temporal resolution of the simulation. Inhibitory inputs were delayed by 1 ms compared with excitatory inputs. The nicotinic synaptic input to SBC was simulated by an additional conductance point source connected to the soma of the SBC model. Parameters of the nicotinic mechanism were extracted from the following *in vitro* recordings: rise time, 83 ms; peak amplitude, 2 nS; decay time constant, 461 ms; E_rev_, 0 mV (data for E_rev_ not shown). An additional onset delay of 10 ms compared with the onset of the auditory nerve inputs was applied to the nicotinic input, as reported for the latency of the olivocochlear bundle effect (Brown et al., 2003). Simulated sound responses were paired with the nicotinic input. The muscarinic modulatory effect on the resting membrane potential was simulated by depolarizing or hyperpolarizing the reversal potential of the leak conductance to −55 mV (simulating the +ACh condition) or −75 mV (simulating the −ACh condition with atropine). Note that no onset or offset dynamic of the modulatory effect was included in the model; thus, all simulations of modulatory effects focus on reviewing the impact of the fully developed phenomenon on the SBC function.

Spike arrival times for the primary inputs driving the excitatory and inhibitory synaptic mechanisms were generated with an inner ear model (Zilany et al., 2014) implemented in the Python module “cochlea” (Cochlea: inner ear models in Python, https://github.com/mrkrd/cochlea, version 1.2). Pure tones of 500 ms duration (including 2 ms cos^2^ ramps) were presented repetitively (100×) at various sound pressure levels (−10 to 75 dB SPL) and frequencies (125–4000 Hz) to the inner ear model. A set of AN output spikes for high spontaneous rate ANFs (“cat” parameters) were generated at a temporal resolution of 10 µs. Low-frequency ANFs (characteristic frequency CF = 1200 Hz) were routinely simulated. For the generation of a peristimulus time histogram (PSTH), higher-frequency ANFs (CF = 3 kHz and tones >3000 kHz) were simulated to prevent effects of phase locking. The same set of auditory nerve responses was used for the different experimental conditions to facilitate comparison of the cholinergic effects. SBC spikes and failures were detected and waveforms of events were analyzed, as previously published (Kuenzel et al., 2011, 2015). Using the peak of the SBC action potential as the spike time, we constructed rate-level functions, PSTHs, and cycle histograms. Temporal precision was quantified with the vector strength of phase locking (Goldberg and Brown, 1969), and a Rayleigh criterion of *p* < 0.001 was used.

All data are presented as the mean ± SEM, unless indicated otherwise. Statistical significance of the influence of the different pharmacological treatment conditions was tested with the Kruskal–Wallis test with Bonferroni-corrected *post hoc* testing (except for the dataset where paired *t* tests were used, see Fig. 5).

## Results

*In vitro* recordings in acute parasagittal slices were performed at the rostral pole of the gerbil AVCN (P14–P25), where the low-frequency SBCs are located (Oertel, 1983; Wu and Oertel, 1984). During the experiment, SBCs were identified by their large, round somata visualized by the IR-DIC optic of the microscope and additionally verified *post hoc* in a subset of recorded cells (*n* = 26) by images showing the somata and the typical bush-like dendritic morphology in biocytin–streptavidin staining (Fig. 1A). In addition, the identification of SBCs was augmented by the following electrophysiological features: (1) during suprathreshold depolarizing current injections, they generate only one (or two) action potentials; and (2) the membrane voltage shows a prominent voltage sag upon steady hyperpolarizing current injection (Fig. 1B). Furthermore, synaptic stimulation of the AN elicited large, depressing EPSCs in SBCs (Fig. 1C). The basic biophysical properties (*R*_m_ = 95 ± 6 MΩ, membrane capacitance C_m_ = 24.4 ± 2.3 pF, *n* = 78) were consistent with previously reported data (Oertel, 1983; Schwarz and Puil, 1997). The average resting membrane potentials of the SBCs were −57.8 ± 0.9 mV (*n* = 78; uncorrected).

### Carbachol elicits a transient nicotinic inward current in spherical bushy cells

To assess the cholinergic responses of SBCs, carbachol was puff applied to the cell in current-clamp recordings. Fifty-five percent of tested SBCs (23 of 42) exhibited some form of transient depolarization. We quantified this in cells where a double-exponential fit of the decay waveform yielded a good match with the actual measurements (r^2^ > 0.8; see Materials and Methods) and found on average a depolarization of 3.7 ± 0.5 mV (Kruskal–Wallis test: *p* < 0.05, df = 29, χ^2^ = 20.05, *n* = 13; *post hoc p* < 0.01 vs ACSF control) relaxing back to the resting potential with a time constant of 398 ± 298 ms (Fig. 1Di). Carbachol puffs in the presence of D-TC, a general blocker of nicotinic receptors, yielded no depolarization (0.1 ± 0.1 mV, *n* = 8, *p* < 0.01 vs carbachol condition), indicating the specificity of the response (Fig. 1Dii). To further explore the composition of the nicotinic receptors present on SBCs, we used MLA, a blocker specific to the α7 subunit containing nAChRs (Fig. 1Diii). Also in the presence of MLA, the carbachol-triggered depolarization was completely abolished (0 mV, *n* = 4, *p* < 0.01 vs carbachol condition). Puff application of ACSF alone yielded no response (Fig. 1Div; 0 mV, *n* = 5, *p* < 0.001 vs carbachol condition). Population data for the depolarization are shown in Figure 1E.

In voltage-clamp recordings at a holding potential of −60 mV, puff application of carbachol into SBCs elicited inward currents (−73.5 ± 22.7 pA; Kruskal–Wallis test: *p* < 0.05, df = 33, χ^2^ = 25.94, *n* = 19; *post hoc* test, *p* < 0.001 vs ACSF control; Fig. 1Fi), which relaxed back to rest with a time constant of 461 ± 117 ms and had a reversal potential of >0 mV (data not shown). The differences in decay time constants derived from the two recording methods are not statistically significant. The peak inward current corresponds to a peak conductance of 1.2 ± 0.4 nS. The washin of D-TC completely abolished the inward current (−1.7 ± 0.6 pA, *n* = 7, *p* < 0.001; Fig. 1Fii), as did the washin of MLA (−0.2 ± 0.2 pA, *n* = 4, *p* < 0.001; Fig. 1Fiii). The specificity of the response was again shown by the fact that puffing ASCF alone yielded no inward current (−0.5 ± 0.3 pA, *n* = 4, *p* < 0.01; Fig. 1Fiv). The population data for the transient inward current are shown in Figure 1G. Combined, these results show that, similar to T-stellate neurons in the AVCN (Fujino and Oertel, 2001), SBCs express functional nAChRs that mediated depolarizing inward currents with slow temporal characteristics when compared to the excitatory glutamatergic auditory nerve fiber input (Isaacson and Walmsley, 1996; Gardner et al., 1999; Xie and Manis, 2013). The complete block of response by MLA suggests that, unlike stellate neurons (Fujino and Oertel, 2001), SBCs exclusively express an α7-containing subtype of the nAChR.

### Differential distribution and subcellular localization of cholinergic transmission in the VCN of the gerbil

**Figure 2.**
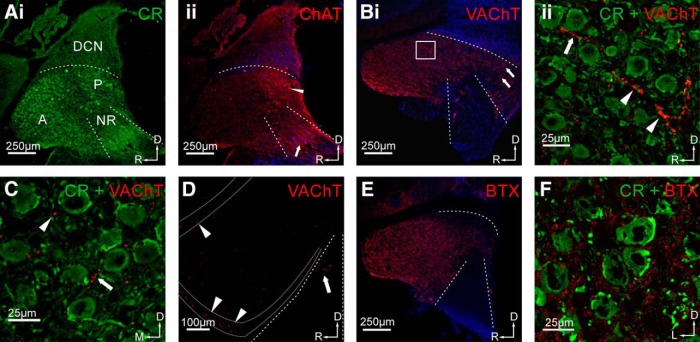
Cholinergic innervation of the CN of the gerbil. ***Ai***, CR staining was used to distinguish CN subdivisions (A = AVCN, P = PVCN, NR = nerve root). SBCs can be indirectly identified by visualization of their large somatic, CR-positive synaptic endbulb of Held terminal. ***Aii***, Staining with anti-ChAT showed immunolabeling in all subdivisions of the CN, with increased signal intensity in the PVCN (arrowhead) and immunolabeling of cholinergic fibers arranged in stripes in the auditory nerve region (arrow). Scale bar, 250 µm. ***Bi***, Staining with antibodies against the VAChT showed strong immunolabeling in the AVCN and PVCN. This staining also revealed VAChT-positive fibers with en passant swellings (white box in AVCN; arrows in PVCN). ***Bii***, The *z*-projection of a high-magnification confocal stack recorded at the site indicated by the white square in ***Bi***, double staining with CR and VAChT. VAChT-positive fibers (arrow) pass through the neuropil between SBCs forming en passant swellings (arrowheads). Scale bars: ***Bi***, 250 µm; ***Bii***, 25 µm. ***C***, Confocal image of a frontal section of the AVCN, double stained with CR and VAChT. In this cutting plane, only few tangentially oriented labeled fibers were observed (arrow); instead, single puncta-like signals were present. Scale bar, 25 µm. ***D***, VAChT staining in a sagittal VCN section with demarcation of the granular cell domain (region between dotted lines). Note the stronger VAChT signals in GCD (arrowheads) compared with the rest of the AVCN. VAChT signals can also be seen in the auditory nerve root (arrow). Scale bar, 100 µm. ***E***, Staining with Alexa Fluor-conjugated α-BTX yielded homogeneous distribution of BTX signals in the AVCN and the region of the auditory nerve root being left blank. ***F***, High-magnification confocal image of CR and BTX double staining in the AVCN showed punctate signals in the neuropil surrounding the SBC. Scale bars: ***i***, 250 µm; ***ii***, 20 µm. Orientation of slices: D = dorsal, R = rostral, M = medial, L = lateral (applies to all images).

In order to confirm the presence of cholinergic innervation in the CN of the gerbil and to determine the distribution of putative cholinergic synapses within the nucleus, immunohistochemical staining was performed in sagittal and frontal slices of the CN in brains prepared from gerbils of age P18–P31. Fluorescent labeling of CR (Fig. 2Ai,Bii,C,F, green) and DAPI (Fig. 2Aii,Bi,E, blue) was used as a reference to visualize the different subnuclei of the CN and to identify the SBCs by the large, CR-positive somatic endbulb terminals.

To visualize the en route cholinergic fibers in the CN, we used an antibody against ChAT. To show presynaptic structures of the cholinergic system, we used an antibody against the VAChT. Staining against ChAT revealed punctuated immunosignals in the neuropil throughout the whole CN (Fig 2Aii), with a higher signal intensity in the posteroventral cochlear nucleus (PVCN) than in the rest of the CN (Fig. 2Aii, arrowhead). Staining arranged in stripes was also seen in the auditory nerve (Fig. 2Aii, arrow), possibly representing cholinergic axons innervating the cochlear root neurons (Gómez-Nieto et al., 2013) or cholinergic collaterals entering the CN via this route. However, we cannot fully explain the staining pattern seen in the nerve root at this moment. VAChT-positive immunosignals were found throughout the entire VCN (Fig. 2Bi), and the VAChT antibody also reliably labeled cholinergic terminals (Fig. 2Bii, magnification of the area marked by the white box in Fig. 2Bi). The VAChT staining revealed both VAChT-positive fibers taking a rostrocaudal course (arrow) and en passant swellings (arrowheads), showing presynaptic cholinergic structures in the immediate vicinity of SBCs. These structures were also found in the PVCN (Fig. 2Bi, arrows). In frontal sections of the AVCN, the VAChT-positive fibers (arrow) were less numerous (Fig. 2C), suggesting a mostly rostrocaudal course of these fibers. VAChT-positive immunosignals were also regularly found in the granular cell layer of the CN [Fig. 2D, dotted lines; granule cell domain (GCD)], with the signal strength even surpassing that in the SBC domain of the AVCN. This is in agreement with previous reports of cholinergic innervation of the CN-GCD (Brown et al., 1988) together with inputs from nonauditory sources (Gómez-Nieto and Rubio, 2009; Zeng et al., 2012). VAChT-positive immunosignals were also observed in the area of the ANR (Fig. 2D, arrow), which is consistent with previous studies showing cholinergic innervation of ANR neurons in rats (Gómez-Nieto et al., 2013).

Postsynaptic sites associated with cholinergic transmission were identified by fluorescently conjugated α-BTX, which specifically binds to nAChR. An overview of the VCN shows strong staining of AVCN and somewhat weaker staining of the PVCN (Fig. 2E). No immunosignals were found in the auditory nerve region, which can be regarded as additional confirmation for the specificity of the α-BTX labeling. In high-magnification confocal images of the AVCN (Fig. 2F, frontal section), the α-BTX immunosignals appeared to be located mostly in the neuropil surrounding SBCs, but rarely in close association with SBC somata. These signal locations indicate a potential dendritic expression of nAChR in the SBC. Especially in low magnification, the “postsynaptic” α-BTX labeling appears stronger and more diffuse than the “presynaptic” ChAT/VAChT signals, which might indicate focal versus widely distributed expression of the respective target proteins and suggests volume transmission. Together, our histological findings agree with the suggestion that the cholinergic inputs enter the rostral AVCN in a caudorostral direction and tend to target the dendritic region of SBCs rather than their somata.

### Muscarinic acetylcholine receptors mediate a long-lasting RMP modulation

**Figure 3.**
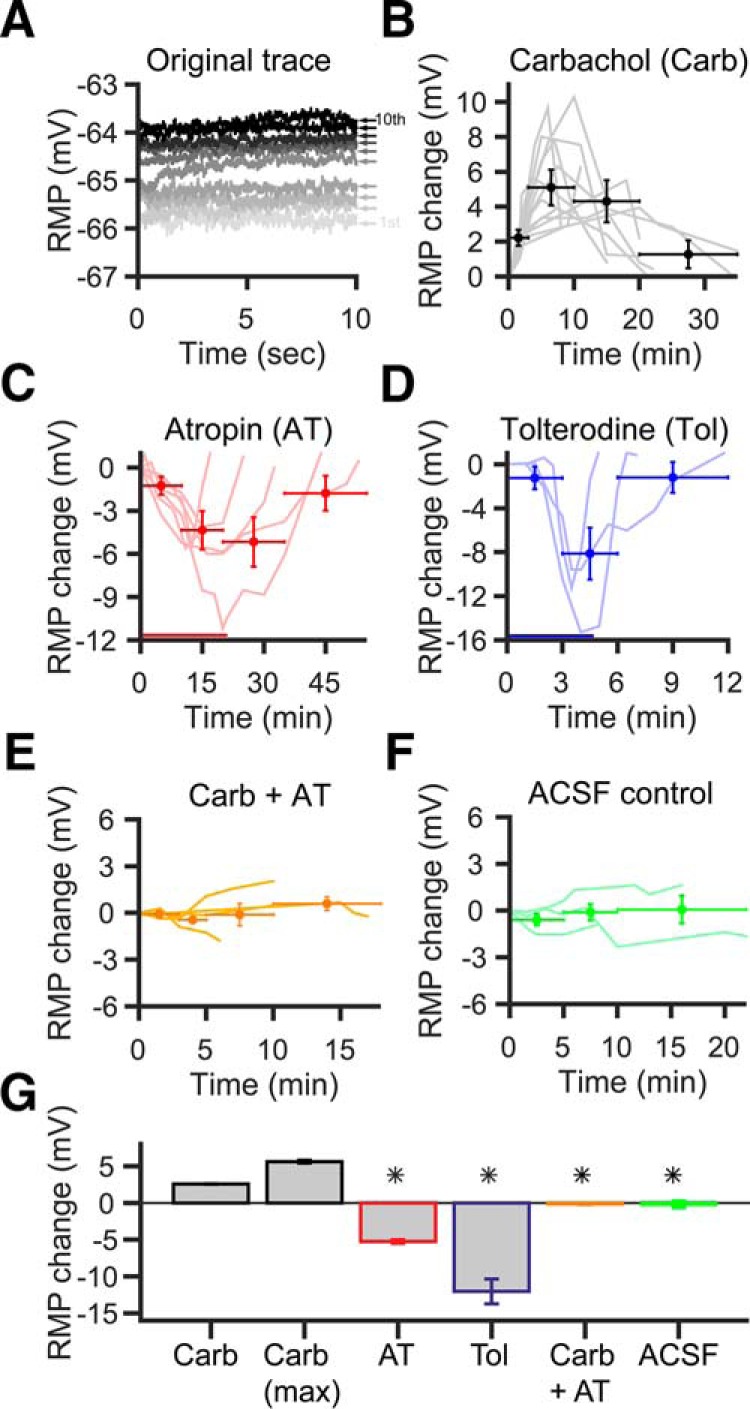
Acetylcholine sets the SBC RMP through muscarinic receptors. ***A***, Representative example trace of SBC depolarization during puff application protocol (see Materials and Methods). The darker the trace, the later the recording took place. Small numbers and the arrow right of the traces indicate puff number. Note the persistent elevation of the RMP, which tends to increase with repeated puff applications. ***B***, Course of RMP changes after 10 carbachol puff applications (0.1 Hz). Gray lines show the RMP courses of single cells (*n* = 13). Circle markers show time-binned RMP averages of all cells: The horizontal error bars indicate the used time interval to calculate the mean, and the vertical error bars denote SD (middle of the crosshair). ***C***, Washin of 2 µm AT (red bar) strongly hyperpolarized the cells. This effect was reversible, and RMP returned to baseline values after washout of AT (*n* = 6, light red lines). Circle markers and error bars show interval and SD of time-binned RMP averages. ***D***, Washin of the specific mAChR blocker Tol (100 nm, blue bar) showed a similar effect as AT washin (light blue lines, *n* = 3); crosshairs show intervals and SD of time-binned RMP averages. Note that differences in the time course of atropine vs tolterodine effects are due to the different perfusion systems used. ***E***, Carbachol application under atropine block. No depolarization occurred when mAChRs were blocked. Light orange lines, single cells (*n* = 5); circle markers, time-binned averages ± SD of all cells. ***F***, Control experiment with application of the vehicle only (ACSF). No depolarization was visible after application. Light green lines, single cells (*n* = 3); circle markers, time-binned average of all cells ± SD. ***G***, Population data for all cells. Bars show the mean of the maximum RMP change. *Significant difference from carbachol condition (*p* < 0.01, Kruskal–Wallis test with Bonferroni *post hoc* test).

In addition to the transient depolarization through α7 nAChRs shown above, longer-lasting depolarizations were seen in 76% of the SBCs (32 of 42 cells) upon repeated application of carbachol. Presenting 10 carbachol puffs over a period of 100 s caused an average depolarization of 2.6 ± 0.1 mV (Kruskal–Wallis test: *p* < 0.05, *n* = 32, df = 56, χ^2^ = 43.39; *post hoc p* < 0.001 vs ACSF control; Fig. 3A,G, ACh). After the end of the carbachol application, the resting membrane potential of the SBCs continued to drift to even more depolarized values. The mean maximum shifts amounted to 5.6 ± 0.2 mV (reached after 11 ± 1 min; *p* < 0.001 vs ACSF control, *p* < 0.01 vs ACh 100 s, *n* = 13; Fig. 3B,G, ACh max). Thereafter, the resting membrane potential recovered with a temporal profile similar to that of the initial values (Fig 3B).

To test whether this depolarization is due to the activation of mAChRs, carbachol stimulation was performed following a washin of atropine (2 µm), a general mAChR blocker, and of tolterodine (100 nM), a synthetic high-affinity mAChR blocker. Surprisingly, both blockers caused a reversible hyperpolarization of SBCs, as seen from the average peak effects: atropine by −5.3 ± 0.2 mV (*p* < 0.001 vs ACSF control, *n* = 6; Fig. 3G); and tolterodine by −12.1 ± 1.6 mV (*p* < 0.001 vs ACSF control, *n* = 3; Fig. 3C,G). This suggests a tonic activation of muscarinic receptors contributing to the SBCs RMP *in vitro*. After a new stable hyperpolarized RMP was reached following either of the two pretreatments, the puff application of carbachol caused no significant SBC depolarization anymore (−0.1 ± 0.1 mV, *p* < 0.001 vs ACh max, *n* = 5; Fig. 3E,G). When ACSF was puffed as a control, no depolarization was noticeable, either after 100 s (−0.1 ± 0 mV, *n* = 3) or after 20 min (−0.2 ± 0.4 mV, *n* = 3; Fig. 3F,G). However, SBCs still showed the nicotinic depolarization in the presence of atropine (2.6 ± 1 mV, *n* = 3; data not shown). Furthermore, the muscarinic depolarization was visible in the presence of D-TC or MLA (2.3 ± 0.3 mV after 100 s, *n* = 6; data not shown).

**Figure 4.**
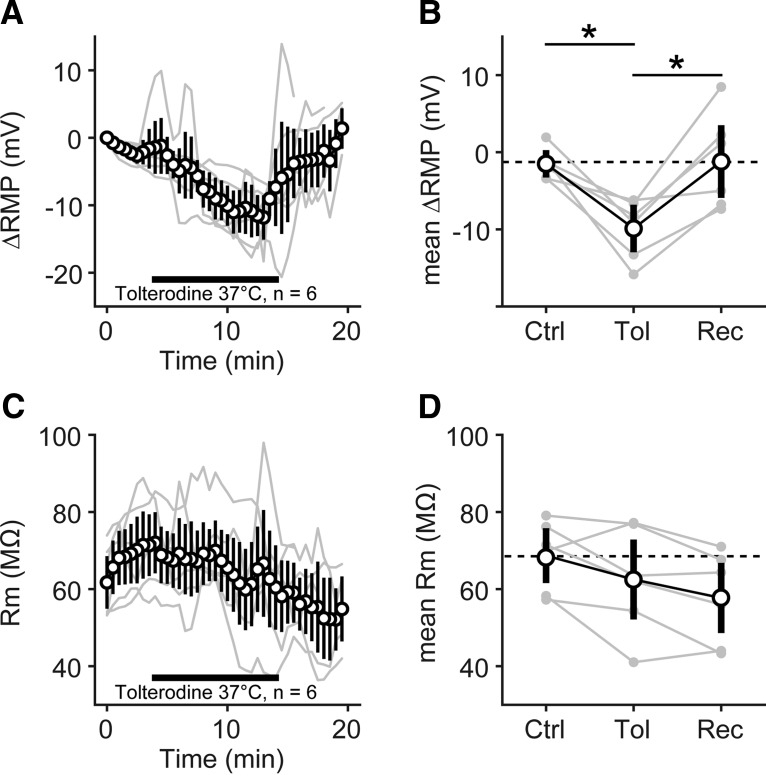
Pharmacological block of muscarinic AChR hyperpolarized the resting membrane potential of SBC at physiological temperature. ***A***, Resting membrane potential of *n* = 6 SBCs (thin gray lines) monitored for 20 min at 37°C. Black open circles and black lines: mean ± SEM measured at 30 s intervals; horizontal black bar, interval of tolterodine washin. ***B***, Averaged RMP before [control (Ctrl), 0-4 min], during (Tol, 4-14min) and after [recovery (Rec), 14–20 min] washin of tolterodine. Gray markers connected by gray lines show individual SBCs; black open circle markers show mean ± SD of *n* = 6 SBCs. Black lines and asterisk show significant differences in *post hoc* testing, *p* < 0.05. ***C***, *R*_m_ of *n* = 6 SBCs from the same measurements and same presentation as in ***A***. ***D***, Averaged *R*_m_ before, during, and after washin of tolterodine, with same presentation as in ***B***.

The majority of our *in vitro* experiments was performed at room temperature (∼23°C). To rule out any adverse effect of the low temperature, especially on G-protein-mediated processes, we repeated the washin experiments with tolterodine at physiological temperature (37°C; Fig. 4). Here, we monitored the change in SBC resting membrane potential and membrane resistance before, during, and after washin of tolterodine. Similar to the experiments at room temperature, we observed a hyperpolarization of the RMP of −9.9 ± 3.8 mV (average of 10 min of washin; *n* = 6; Fig. 4A,B). The average RMP during washin of tolterodine was significantly different from the average RMP before and after washin (Kruskal–Wallis test: *p* < 0.05, df = 17, χ^2^ = 9.1; *post hoc U* test, *p* < 0.05 Tol vs control and Tol vs recovery; *p* = 0.94 control vs recovery). Surprisingly, we did not observe a consistent change in *R*_m_ during the washin of tolterodine (Fig. 4C), possibly due to the comparably large variability given the low absolute *R*_m_ value of the SBCs. Although *R*_m_ slightly diminished upon washin (control, 69 ± 9 MΩ; tolterodine, 62 ± 14 MΩ), this difference was not statistically significant (Kruskal–Wallis test: *p* = 0.31, df = 17, χ^2^ = 2.3) and furthermore did not recover (recovery, 58 ± 12 MΩ) after switching the perfusion back to ACSF.

Thus, in addition to (and independently of) the transient depolarization mediated by nicotinic AChR, the RMP of SBCs is slowly shifted to depolarized values by carbachol through the action of muscarinic AChR. This mechanism is independent of temperature and appears tonically active in SBCs. Thus, mAChRs critically contribute to determining the RMP of the SBC.

### The cholinergic RMP depolarization increases the spiking probability of SBCs *in vitro*

**Figure 5.**
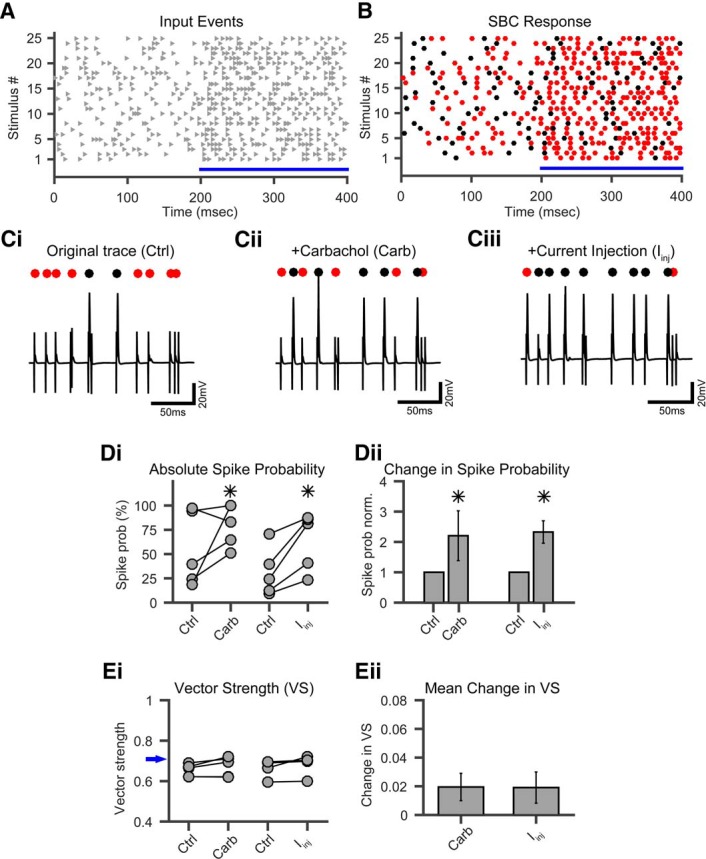
Cholinergic modulation increases SBC spike probability *in vitro* without a loss in temporal precision. ***A***, Twenty-five repetitions of IVL stimuli were successively presented with a 3 s interval. Gray triangles, input spikes. ***B***, Example response of one SBC to the IVL stimulus paradigm. Red dots, Failed AP; black dots, successful AP. ***C***, Excerpts from IVL stimulus 18 under control conditions (***Ci***), under cholinergic modulation via carbachol (Carb; ***Cii***), and with elevated RMP through *I*_inj_ (***Ciii***). Note the increase in spike probability. Markers, Red dots, failed AP; black dots, successful AP. Calibration: 20 mV and 50 ms. ***D***, Increase in spike probability under cholinergic modulation. ***Di***, Absolute change in spike probability for cholinergic modulation (Carb) and for RMP elevation through *I*_inj_. Asterisks indicate significant difference (*p* < 0.01, paired *t* test). ***Dii***, Mean relative change in spike probability for all cells in ***Di***. Spike probability is increased twofold compared with control for both conditions. Asterisks indicate significant difference (*p* < 0.01, paired *t* test). ***E***, Changes in vector strength under cholinergic modulation and RMP elevation. ***Ei***, No significant change in absolute VS of SBC output for cholinergic modulation or RMP elevation was observed (*p* > 0.7, paired *t* test). Blue arrow denotes input-VS from the IVL stimuli. ***Eii***, Only a slight, but not significant (*p* > 0.7, paired *t* test), increase in mean VS could be seen for both conditions.

Next, we tested whether the carbachol-induced depolarization affects the probability of AP generation and also the temporal precision of the SBC spiking. For this, minimal electrical stimulation of ANF inputs to SBCs was paired with the puff application of carbachol. For the synaptic stimulation, IVL temporal stimulation patterns were used to mimic spontaneous and sound-driven activity by high-spontaneous rate ANFs (Fig. 5). This resulted in an SBC spike probability of >20%. If an SBC had a spiking probability of 0% (i.e., showed only EPSPs) or 100% (showed only action potentials), they were not included in this analysis because of the limited dynamic range on either end. If a stable synaptic stimulation was achieved and the control spike probability was established, we applied our carbachol stimulation paradigm (10 puffs, 0.1 Hz). When the cells showed a clear cholinergic reactivity, we repeated the synaptic stimulation with an unchanged stimulus intensity. Under carbachol influence, the cells showed a significant increase in spiking probability (paired *t* test, *p* < 0.01, *n* = 5; Fig. 5Cii,Dii, ACh). To test whether the increased spike probability was directly caused by the carbachol-mediated RMP elevation, we paired the synaptic stimulation with an RMP elevated by 5.5 mV through current injection in a different set of cells. In these experiments, we also observed a significant increase in spike probability (paired *t* test, *p* < 0.01, *n* = 5). It increased to a similar extent as it did for the cholinergic modulation [Fig. 5Ciii,Di, current injection (*I*_inj_)], although a direct statistical comparison between the carbachol application and current injection was not performed. Because the SBCs showed a large variability in their spike probability under control conditions, we calculated the mean relative increase for all paired recordings, showing that the spike probability significantly increased more than twofold for both treatment conditions (ACh, 2.2 ± 0.9-fold increase; RMP, 2.3 ± 0.4-fold increase; paired *t* tests, *p* < 0.01; Fig. 5Dii).

Because the temporal precision is one of the prime characteristics of binaurally converging input to the medial superior olive, we explored *in vitro* how the carbachol-induced increase in SBC AP probability influences the timing of APs. The temporal AP jitter, quantified as the average SD of the AP latency, decreased from 0.32 ms (control conditions, *n* = 5 for each condition) to 0.17 ms in carbachol-treated cells (*n* = 5), and to 0.17 ms (*n* = 5) in cells that were depolarized through current injection; however, did not reach statistical significance in both cases (data not shown). For the carbachol treated cells, mean VS was 0.67 ± 0.04 (*n* = 5) compared with 0.65 ± 0.03 (*n* = 5) under control conditions (Fig. 5Ei,ii). When SBCs were depolarized through current injection, the initial VS values were 0.66 ± 0.04 (*n* = 5) and increased to 0.69 ± 0.05 (*n* = 5) for elevated membrane potentials (Fig. 5Ei,ii). Due to the paired paradigm, we did not test whether VS differed for carbachol treatment versus current injection. The input VS of the IVL stimuli was 0.7 in all experiments. On average, the mean absolute change in vector strength was minimally positive and, furthermore, not statistically significant. Together, we found the vector strength to remain stable in cells subjected to carbachol-induced depolarization or direct depolarization through current injection (Fig. 5Ei).

In summary, the action of mAChRs increased the spike probability *in vitro* by bringing the SBCs closer to their firing threshold, but at the same time did not interfere with the temporal precision of spike timing. These data suggest that the muscarinic component of the cholinergic modulation causes a twofold increase in well timed output spikes without a reduction in temporal precision (i.e., the synchronization rate of the SBC output is substantially increased by the action of the cholinergic top–down system).

### Carbachol increases the SBC spiking rate *in vivo*

**Figure 6.**
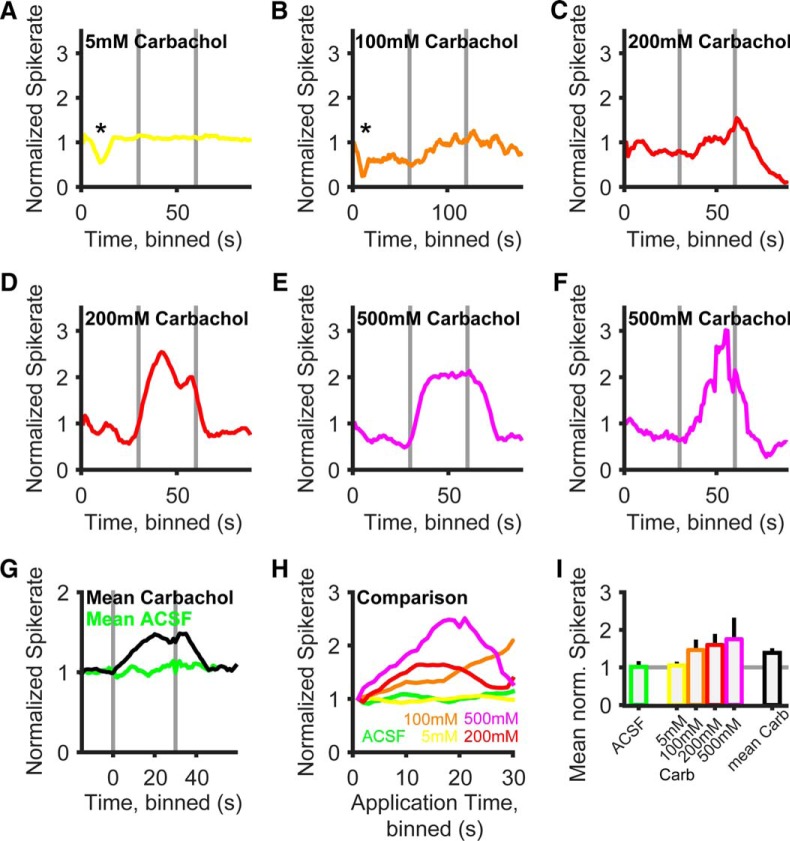
Carbachol increases SBC spike probability *in vivo*. ***A–F***, Example recordings of spontaneous spike rate in six different identified SBC units *in vivo* at four different carbachol concentrations (5**–**500 mm). Horizontal gray bars mark onset and offset of iontophoretic application. Spike rates are normalized to the average of the first 5 s of spontaneous activity. Asterisks in ***A*** and ***B*** mark a reduction in the spontaneous spike rate by glycine control iontophoresis to 55% in ***A*** and 24% in ***B***. ***G***, Grand average of spontaneous spike rate profiles over all units and carbachol concentrations (black line, n = 24), aligned on the onset of the iontophoretic current. Carbachol application resulted in an increase in spiking activity, while ACSF application had no effect (green line, n = 7). ***H***, Mean rate increase and temporal profile of spike rate is dependent on the concentration of the iontophoretic drug. Superimposed spike rate profiles for the different concentrations shown in the same colors as ***A–F***; the mean spike rate profile under ACSF iontophoresis is shown in green. Note that for increased visibility only the segments during ionotophoretic application are shown here (0 s = start of iontophoresis). ***I***, Comparison of spontaneous spike rates during iontophoretic drug application plotted as the mean ± SD (colors same as ***A–H***).

Next we tested whether the increase of spike probability caused by the influence of cholinergic inputs on the RMP can also be observed in the intact brain of anesthetized gerbils. To this end, single-unit recordings were acquired from a total of 24 identified spherical bushy neurons in the rostral AVCN of gerbils in the age range P22 to P38, while iontophoretically applying carbachol (Fig. 6). When using a low concentration of carbachol (5 mm; Fig. 6A), none of the units (*n* = 4) tested responded with a change in spontaneous firing rate. The spontaneous rate profile over the 30 s of iontophoresis was comparable to that of the control ACSF application (Fig. 6H), and the average rate was not statistically different from that of negative controls (*p* = 0.38, 105 ± 4% for 5 mm carbachol vs 101 ± 10% for ACSF control). Iontophoretic application of glycine, however, transiently reduced the spontaneous firing rate (Fig. 6A, asterisk) in the same units, indicating the responsiveness of the spontaneous firing rate in these units to iontophoresis of pharmacological agents. No significant changes (*p* = 0.63) in SBC spontaneous firing occurred upon iontophoresis with ACSF (*n* = 7; Fig. 6G, average response to ACSF). Higher concentrations of iontophoretically applied carbachol (100 mm, *n* = 9; 200 mm, *n* = 7; 500 mm, *n* = 4) resulted in a significant deviation of the spontaneous firing rate. Iontophoresis of 100 mm resulted in a slow and delayed rise in spontaneous action potential firing (on average to a maximum of 200% after 30 s; mean rate, 146 ± 22%) that gradually returned to baseline after the end of the iontophoretic currents (Fig. 6B). Higher concentrations of carbachol (Fig. 6C–F) caused on average a more rapid increase in spontaneous SBC firing (Fig. 6H) and a higher average spontaneous spike rate during drug application (Fig. 6I). At 200 mm, a maximal increase to 160% after 12 s was observed (mean rate, 159 ± 24%); at 500 mm, the spontaneous spike rate was increased to a maximum of 250% after 21 s (mean rate, 175 ± 68%). However, in a number of cases a reduction of spontaneous firing rates followed the initial intense increase of spontaneous spiking (Fig. 6D,F) during the ongoing iontophoretic drug application. This resulted in a phasic-like response to the carbachol iontophoresis and was quite common in the two highest concentrations, as can be seen from the average response profiles per concentration (Fig. 6H), but never occurred with 100 mm carbachol. On the other hand, even at 500 mm carbachol the reduction of spike rate was absent in one unit (Fig. 6E). In all cases, this effect could be distinguished from a reduction of measured spike rates caused by pure technical reasons (e.g., “losing the unit”; Fig. 6C, see end of recording), because the reduction of spiking caused by the iontophoretic drug application recovered to the steady-state spontaneous firing rate after some time or did not go below this value at all. At this moment, we cannot exclude unspecific responses to the iontophoresis (e.g., effects of pH) as an explanation for the reduction of the spontaneous rate in some of the SBC upon strong carbachol iontophoresis.

The mean effect of the iontophoretic application of carbachol, averaged over all of the SBCs and the concentrations (*n* = 24 SBC, *n* = 4 concentrations) used, is shown in Figure 6G. Over all units, carbachol iontophoresis causes a significant increase in spontaneous spike rate to 148% after 20 s (mean, 139 ± 7%, *p* < 0.01). After the end of the iontophoretic current, the spontaneous spike rate reached baseline values after 16 s. Together, a significant increase in spontaneous spike rate was seen upon carbachol application to SBCs *in vivo*. In this set of *in vivo* experiments, we did not apply sound stimulation and therefore cannot yet comment on the functional impact of cholinergic modulation on temporal coding and signal processing. It is nevertheless evident that in the intact adult gerbil brain cholinergic responses of SBCs cause a slow and sustained increase in resting spike probability that is consistent with our observations in acute brain slice experiments.

### Functional relevance of the cholinergic modulation of SBC excitability

**Figure 7.**
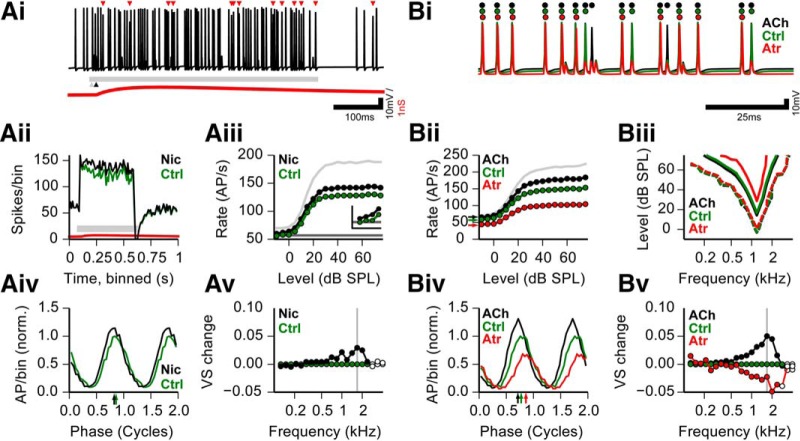
*In silico* model of the functional relevance of cholinergic SBC modulation. ***Ai***, Example trace of simulated SBC membrane potential, including sound-driven excitation and inhibition, and matched nicotinic input. Calibration: 100 ms and 10 mV. Red triangles, Extra action potentials that failed in the simulation of the same spike train without the nicotinic input; red trace, shape of the nicotinic PSP (the *y*-scale represents 2 mV for this trace); gray bar, sound stimulus; black arrowhead, onset of nicotinic event. ***Aii***, Peristimulus time histogram for 100 repetitions of Ai with (black line) and without (green line) nicotinic input. Gray bar depicts stimulus duration, and red line depicts nicotinic PSP, as in ***A***. Simulation conditions were 3 kHz, 20 dB SPL; CF = 3 kHz. ***Aiii***, Rate-level function (RLF) for −10 to 75 dB SPL, 3 kHz. CF = 3 kHz. Dark gray line shows identical spontaneous rates for all conditions. Colors are as in ***Aii***. Light gray curve shows the input RLF. Inset shows the first four levels (−10, −5, 0, and 5 dB SPL; scale of inset: *x*-axis, −16 to 6 dB SPL; *y*-axis, 50–70 spikes/s). ***Aiv***, Period histograms of spike times for the nicotinic condition; colors are as in ***Aii***, normalized to the maximal amplitude of the control condition. Arrows show preferred phase. Simulation conditions shown here were 1682 Hz, 60 dB SPL, CF = 1200 Hz (see gray vertical line in ***Av***). ***Av***, Relative change in vector strength to 60 dB SPL tones of different frequencies for nicotinic condition compared with the control condition. Colors are as in ***Aii***. Filled circles depict significant phase locking. Gray vertical line shows the frequency of the period histogram in ***Aiv***. ***Bi***, Example trace of the same spike train simulated at the three modulatory conditions. Red, simulated atropine; black, simulated carbachol; green, control. Circles mark the occurrence of action potentials under the three conditions. Calibration: 25 ms and 10 mV. ***Bii***, Rate-level functions for the three modulatory conditions in ***Bi***; colors are as in ***Bi***. Light gray curve shows input RLF. Simulation conditions were 1.2 kHz, CF = 1.2 kHz. Arrows show the spontaneous rate per condition. ***Biii***, Frequency response area for 9 stimulus levels and 21 frequencies; colors are as in ***B***. Dashed contour for each color shows +5 Hz increase in response spike rate vs spontaneous rate; inner, solid contour shows +50 Hz increase in response spike rate vs spontaneous rate. CF = 1.2 kHz. ***Biv***, Period histograms of spike times for the three modulatory conditions; colors are as in ***Bi***, normalized to the maximal amplitude of the control condition. Arrows show the preferred phase. Simulation conditions were 1682 Hz, 60 dB SPL, CF = 1200 Hz. ***Bv***, Relative change in vector strength to 60 dB SPL tones of different frequencies for three modulatory conditions, compared with the control condition. Colors are as in ***B***. Filled circles depict significant phase locking. Gray vertical line shows the frequency of the period histogram in ***Biv***. Ctrl, Control; Nic, nicotinic input.

We next assessed the potential functional impact of the cholinergic inputs to SBCs in the context of the auditory sensory role of SBC using an *in silico* approach. The *in silico* approach was chosen (1) because our *in vivo* experiments lack responses to acoustic stimulation, and (2) to differentially analyze the interaction of sound stimulation with the nicotinic transient and the muscarinic modulatory effect. In an SBC model that was used in previous studies (Kuenzel et al., 2011, 2015; Nerlich et al., 2014a), we implemented the transient nicotinic input as a weak (2 nS), long-lasting (rise time, 83 ms; decay time constant, 461 ms) depolarizing cation conductance (E_rev_ = 0 mV). When paired with excitatory inputs driven by simulated auditory nerve inputs (Fig. 7Ai, auditory stimulus indicated by horizontal gray bar) the nicotinic postsynaptic potential (PSP) caused an increase in P_AP_ in the SBC model. Since we imposed a 10 ms delay of the nicotinic event with respect to the sound response onset, the increase in P_AP_ is especially pronounced in the early ongoing phase of the sound stimulation, as can be seen in average peristimulus time histograms (Fig. 7Aii, green, control without nicotinic input; black, with additional nicotinic input). During this segment of the response, the number of failures due to the accumulated effects of refractoriness and the impact of acoustically evoked inhibition is usually increased (Kuenzel et al., 2011; Keine and Rübsamen, 2015). The additional depolarization caused by the nicotinic event, however, sufficed to push a significant number of events back above threshold. Analyzed over many stimulus levels, this effect was more pronounced at higher response rates (i.e., higher SPL; Fig. 7Aiii). Nevertheless, also for very low SPLs, the nicotinic modulation increased the signal-to-noise ratio between stimulus responses and spontaneous baseline (Fig. 7Aiii, inset).

Next, we simulated the fully developed muscarinic modulation as a shift of the reversal potential of the leak conductance. Given the results of the *in vitro* experiments (Figs. 3–5) and our inability to extract a consistent muscarinic conductance change from our data, we deemed this a sufficiently good “first-order” approximation of the observed muscarinic effect. The simulated RMP changes affected P_AP_, especially during higher firing rates (i.e., short interspike intervals). The simulated block of nicotinic RMP elevation by atropine (E_rev_ = −75 mV; Fig. 7Bi, red) resulted in more failures than under control conditions (Fig. 7Bi, green). Furthermore, during the simulated carbachol condition (E_rev_ = −55mV; Fig. 7Bi, black), even fewer failures occurred. In the simulated atropine condition, a higher number of failures occurred, and the effects of refractoriness at higher stimulus levels (Fig. 7Bii) were more pronounced than for the control condition (E_rev_ = −65 mV). The impact of the muscarinic modulation became most obvious during the rising slope of the rate-level function (0–20 dB; Fig. 7Bii; i.e., the dynamic response range of the unit). Similar to the nicotinic effect, the simulated carbachol condition (E_rev_ = −55 mV) resulted in a further elevation of P_AP_ compared with the control condition. In contrast to the nicotinic event, the modulatory effect did indeed increase spontaneous firing rates as well, as seen in the *in vivo* recordings (compare Fig. 6, 7Bii, arrows). When we simulated a complete frequency response area for each condition (Fig. 7Biii), we saw that the general shape of a contour where the response was close to threshold (5 spikes/s elevated over the spontaneous response, dotted lines), was indistinguishable between conditions. Thus, for low stimulus levels the modulatory effect does not enhance the signal-to-noise ratio. However, the response width of the simulated atropine condition markedly deteriorated with higher response levels, as can be seen for the 50 spikes/s elevation contour (but see Fig. 7Bii, solid lines). Thus, in the context of the *in vitro* and *in silico* results, a depolarized RMP caused by moderate ambient ACh levels increases excitability and extends the dynamic range of the tuning of the SBC.

We next investigated whether the two nicotinic effects have an impact on spike time precision and thus the phase coding of the SBC. Therefore, responses to a range of pure tones under the different ACh conditions were simulated. The interaction of excitatory and nicotinic inputs caused an amplitude increase in the period histograms (Fig. 7Aiv) with only very little change in the preferred phase. Over all of the frequencies tested, the nicotinic input resulted in a small increase in vector strength, especially for frequencies >1 kHz (Fig. 7Av). Results were more pronounced for the simulated modulatory effect (Fig. 7Biv,v). Increasing the cholinergic modulation (i.e., depolarizing the RMP) resulted in markedly enhanced period histogram amplitudes, an advance of the preferred phase (Fig. 7Biv), and more pronounced improvement of phase-locking accuracy (Fig. 7Bv). On the other hand, a reduction in cholinergic modulation (i.e., hyperpolarizing the RMP) resulted in reduced period histogram amplitudes, a phase delay (Fig. 7Biv), and impaired phase locking (Fig. 7Bv).

Together, *in silico* experiments suggest that both nicotinic and muscarinic modulation of SBCs increases the output of well timed action potentials and that a baseline of muscarinic activation is necessary to set the working RMP and excitability of SBCs.

## Discussion

Here we propose a novel mechanism for tuning the RMP and spike probability of time-coding sensory neurons in the auditory brainstem of mammals through cholinergic signaling. We showed that the RMP of SBCs is modulated on different time scales through nicotinic and muscarinic AChRs. While the activation of α7-nAChRs transiently depolarized the SBCs with a time constant of several hundred milliseconds, the level of activation of mAChRs dynamically determined the RMP in a range of several minutes. Cholinergic modulation leads to an increased spiking probability *in vitro* by moving the membrane potential closer to threshold. The temporal acuity of the SBC output, however, was stable despite the increase in P_AP_. The *in vitro* data were supported by *in vivo* single-unit recordings combined with pharmacology, where carbachol iontophoresis increased the spontaneous spike activity of SBCs. In agreement with the experimental results, an SBC model that included a first representation of the cholinergic effects predicted an increase in spiking responses, expanding the dynamic sound response range of the neuron and increasing the number of well timed output action potentials. Together, our results suggest top–down control of information processing in monaural time-coding neurons in the initial stages of the auditory pathway mediated by cholinergic modulation.

### The AChR composition of SBCs differs from other cell types in the CN

The existence of cholinergic signaling in the cochlear nucleus has been investigated before for T-stellate cells in the VCN (Fujino and Oertel, 2001), cartwheel and giant cells in the dorsal cochlear nucleus (DCN; Pál et al., 2009; He and Wang, 2014), as well as for cells of the granular cell domain (Irie et al., 2006; Kó́szeghy et al., 2012). Cholinergic signaling onto time-coding neurons like the SBC has not yet been systematically analyzed (but see Oertel and Fujino, 2001; Bledsoe et al., 2009). We found that in SBCs only α7 subunit-containing receptors are expressed. We thus conclude that SBCs solely express the α7 homopentamer nAChR (Happe and Morley, 1998; Yao and Godfrey, 1999a,b; Morley and Happe, 2000; Morley, 2005). Our histological data indicate dendritic localization of the AChR, a feature that has also been shown for other secondary inputs on SBCs (Gómez-Nieto and Rubio, 2009). The α7 nAChR has a high Ca^2+^ permeability (Berg and Conroy, 2002), which could explain the drop in *in vivo* spontaneous spike rate after carbachol application in some of our *in vivo* recordings. Unphysiologically high nicotinic activation could possibly cause Ca^2+^-dependent modifications of potassium or hyperpolarization-activated cyclic nucleotide-gated channels or activation of Ca^2+^-dependent conductance, even though SBCs show strong expression of calcium-binding proteins (Bazwinsky et al., 2008). It can, however, not be ruled out that the reduction of the spontaneous rate represents an entirely unspecific effect of the iontophoresis (Franken et al., 2015).

The sole expression of the α7 receptor subtype is different from that in stellate cells, which express a mixture of α7- and α4β2-containing nAChRs (Fujino and Oertel, 2001). D-stellate cells and octopus cells (Fujino and Oertel, 2001; Oertel and Fujino, 2001), on the other hand, show no AChRs at all. Physiological data for the DCN is restricted to muscarinic receptors, since they account for most of the cholinergic signaling in the DCN (Irie et al., 2006; Pál et al., 2009; Kó́szeghy et al., 2012; He and Wang, 2014). In the DCN, combinations of m1–4 mAChR expression have been reported (Chen et al., 1994; Godfrey et al., 1998; Irie et al., 2006; Pál et al., 2009; Zhao and Tzounopoulos, 2011; He and Wang, 2014). For the AVCN, only the existence of the m2 and m3 subtypes of mAChRs could be shown (Yao and Godfrey, 1995, 1996; Yao et al., 1996; Hamada et al., 2010). Since the m3 receptor mediates postsynaptic excitation by inhibiting potassium currents (Brown, 2010; Haga, 2013), a phenomenon termed the M-current (Brown and Adams, 1980), we hypothesize that the modulation of SBC RMP is caused by m3 mAChR activation. However, since we could show neither a consistent increase nor a decrease in *R*_m_ upon muscarinic activation, the exact mechanism of the muscarinic modulation of SBCs remains to be investigated.

### Endogenous ACh sets SBC RMP

Pharmacological blockade of mAChRs strongly hyperpolarized SBCs. We hypothesize that already at rest SBCs are tonically depolarized by endogenous ACh released in the vicinity of SBCs. This participates in setting the RMP and the excitability of the SBC. The idea of RMP modulation by endogenous ACh has also been proposed for fusiform and cartwheel cells of the DCN (Godfrey et al., 1998). It has also been shown that the SBC RMP is set by endogenous glutamate through mGluRs (Chanda and Xu-Friedman, 2011; Yang and Xu-Friedman, 2015). Although we could represent the muscarinic effect only in a simplified form in the model, some general conclusions can be drawn from our *in silico* experiments. First, as shown by the simulated atropine/hyperpolarized condition, a baseline ACh level seems necessary to achieve a functional excitability and dynamic response range of the model SBC. Second, further cholinergic input (the simulated carbachol/depolarized condition) positively influences SBC P_AP_, especially in conditions with numerous failures and also improves temporal precision.

### Functional implications of different time constants of cholinergic modulation

Although our model of the muscarinic modulation was simplified, and we did not perform combined *in vivo* pharmacology and sound stimulation, our *in silico* results nevertheless suggest that both nicotinic activation and full muscarinic modulation increases P_AP_ and the dynamic range of SBCs. Together with the increase in P_AP_, we found an increase in temporal precision in the model under both nicotinic and muscarinic modulation. The combined increase in rate and precision causes a marked increase in the number of well timed SBC action potentials, which could suffice to improve detection and localization of tones in noise by enhancing interaural time difference cues used for binaural unmasking further up in the auditory pathway. Also, the responses of SBCs to any sound stimulus that occurs temporally aligned with the nicotinic input to SBCs will cause more output spikes compared with unmatched stimuli, without a deterioration of output precision. This is especially pronounced at mid to high SPL, where the additional depolarization by the nicotinic PSP helps to overcome refractoriness and the tonic inhibition (Nerlich et al., 2014a) in the ongoing segment of the sound response. Based on our results, we hypothesize that the nicotinic and muscarinic receptors possibly mediate the same effect at different time scales. This also seems probable with respect to the two time scales that the OCB exhibits. The fast olivocochlear effect works in the range of tens of milliseconds, whereas the slow effect lasts up to minutes (Guinan, 2006). The nicotinic activation of SBCs could provide a fast but phasic reaction that is activated during the onset of the peripheral olivocochlear effect. The muscarinic activation, on the other hand, could provide a long-lasting modulation that is activated during the slow and ongoing olivocochlear effect. The activation of medial fibers of the OCB reduces the gain of the cochlea and enhances the detection of tones in noise (Guinan, 2006), but at the cost of a reduced dynamic range. Neuronal processing in low-frequency spherical bushy cells, which receive only one or very few AN inputs, already imposes a reduction of dynamic range compared with AN inputs due to synaptic properties (Wang and Manis, 2008) and interaction with inhibitory inputs (Kuenzel et al., 2011; Keine and Rübsamen, 2015). The total loss of dynamic response range at the SBC output level during OCB activation could thus impair sound processing downstream of SBCs. However, the increased excitability of cells in the CN caused by cholinergic innervation through collaterals of the OCB, which seem to make up most of the cholinergic innervation in the CN (Mellott et al., 2011), serves to ameliorate the total loss of dynamic range by making SBC input and output more similar.

### ACh modulates auditory processing

In the auditory system, ACh has been implicated in regulating auditory plasticity (Keuroghlian and Knudsen, 2007). In general, widespread modulation of the brain through ACh is implicated in setting the behavioral state of the animal (Lee and Dan, 2012; Aton, 2013). It has been shown that the attention, motivation, and vigilance of the animal, mediated largely by ACh, greatly influence sensory processing (Pepeu and Giovannini, 2004; Hasselmo and Sarter, 2011; Lee and Dan, 2012; Smucny et al., 2015; e.g., decreasing sensory detection thresholds for attended stimuli while increasing thresholds for the unattended stimuli; Aton, 2013). We hypothesize that cholinergic signaling in the AVCN of the gerbil is partially under top–down control from brain areas that are not immediately within the auditory pathway. It has been shown that, apart from OCB input, cholinergic projections from the pontomesencephalic tegmentum enter the CN (Mellott et al., 2011). This nucleus regulates arousal and vigilance, and is under direct cortical control. Also, the OCB signaling is under additional cortical control (Doucet et al., 2002). It is intriguing to speculate that, through ACh, SBC excitability is also modulated in a stimulus-independent, top–down manner. This mechanism may ultimately be regulated by the behavioral state of the animal and goes beyond the already known, stimulus-dependent signaling through OCB collaterals.

## Synthesis

The decision was a result of the Reviewing Editor Leonard Maler and the peer reviewers coming together and discussing their recommendations until a consensus was reached. A fact-based synthesis statement explaining their decision and outlining what is needed to prepare a revision is listed below. The following reviewer(s) agreed to reveal their identity: Yong Lu

The revised manuscript has now been re-reviewed by two reviewers. One reviewer finds that the critiques raised in their previous review (for the Journal of Neuroscience) have been successfully addressed.

The other reviewer is mostly positive and finds that the main issues previously raised have been addressed. However there are still some issues remaining. This reviewer and I agree that the issues mostly concern wording, references and some clarification of technical issues. The revision will therefore not require re-review. As Editor, I will however carefully check that all the criticisms presented below have been carefully addressed.

Please be sure to reply (in your rebuttal) on how you have addressed all the points listed below.

Thank you and congratulations on an important contribution to the field.

Dr. Leonard Maler

Reviewer #1

This revised manuscript addresses a number of the issues raised in the previous review reasonably well. However, there are a number of concerns that remain.

Lines 54, 55: Because the data with in vivo recordings does not address the timing issue, which would require additional in vivo recordings that the authors are not presently able to make, the sentence should be reworded to make it very clear that the the "exquisite temporal acuity" is only supported by in vitro data.

Line 78: This sentence doesn't make a clear statement. "Among these" does not include the cholinergic system (only GABAB and mGluR were raised in the previous sentences), and it is not clear who "discussed" cholinergic connections. Reword: "Other neuromodulatory influences are also present, such as from the cholinergic system, but these have not been as extensively investigated." (although, this is somewhat debatable, given the other cited studies...)

Line 204: provide a valid link for the antibody database.

l338: "Please note: the" should be simply "The ..."

l347: The Bellingham paper seems to have been selected at random. As you are working in gerbil, would not a gerbil paper be more appropriate? What about just noting the time course of the EPSCs in Fig. 1C (do you have enough cells to fit a decay time constant and provide an estimate of variabiity?). Otherwise maybe you should cite other work that was focussed on the time course of the EPSCs (Isaacson and Walmsely, rat; Gardner et al, mouse; Xie and Manis, mouse).

l362-363. I think that the parallel staining in the AN root area is indicative of exclusion of the VChaT-positive fibers from the AN fiber bundles - I'm also actually surprised that this appears so distinctive in the immunstaining. My understanding is that the MOC and LOC efferent fibers exit with the vestibular nerve (not the auditory nerve), and they do not join the auditory portion until they cross over in the anastomosis of Oort much closer to the cochlea. Although it is possible that this is different in the gerbil, so that the fibers run in the central part of the nerve root, it seems unlikely. The statements made here should be modified.

l483-484: This test does not necessarily indicate "functional iontophoresis" from the carbachol barel however.

The last paragraph of the results is really a discussion point and belongs in the discussion. It is also quite unusual in scientific writing to speak directly to the reader as is done on line 572. I suggest rewording this.

l605: This statement is not strictly correct. Oertel and Fujino (2001) have commented somewhat informally on the "subtle" effects of ACh on mouse CN bushy cells. There are also hints from Beldsoe et al (2009). The studies of Comis and Whitfield (1968) are harder to interpret because they did not do an adequate job of unit typing, but they also report some of the fundamental effects.

l609-611: Why do you think a high Ca permeability would produce a drop in spike rate? I don't follow the subsequent argument. Are you thinking of activation of SK channels (leading to hyperpolarization), or Ca-dependent kinase modulation of K or Ih channels (isoforms of PKC; leading to shift in the voltage-dependence of the channels and influencing Rm and spike threshold)?

l641-643: This discussion is pretty "hand-wavy". Please tighten it up or better yet, delete it.

l671-673: If you reduce the peripheral dynamic range, exactly how do you re-expand it in the central system, from a spike-coding standpoint, with the mechanisms you have suggested? How does this notion fit with the changes in SR that you report from the in vivo studies? If you wish to make the suggestion in these lines, I recommend that you more carefully walk the reader through the steps involved. In the model, I see an expansion of the range of spike rates, but not of sensory dynamic range in terms of the RI function. A similar claim regarding dynamic range is made in lines 546-550. This might depend on how one defines the dynamic range, and how it has been used in the past in studies of the OCB effects, so additional precision could be useful here to clarify what you mean, and to more clearly put it in the context of what happens to the ANF input with OCB activation.

The last paragraph of the discussion is so general, and relies on so many assumptions, as to not be informative.

Re previous review: The term "backing current" refers to what you have called "retention current"
